# Anomalous magneto-resistance of Ni-nanowire/Nb hybrid system

**DOI:** 10.1038/s41598-019-50966-8

**Published:** 2019-10-09

**Authors:** O. V. Skryabina, S. N. Kozlov, S. V. Egorov, A. A. Klimenko, V. V. Ryazanov, S. V. Bakurskiy, M. Yu. Kupriyanov, N. V. Klenov, I. I. Soloviev, A. A. Golubov, K. S. Napolskii, I. A. Golovchanskiy, D. Roditchev, V. S. Stolyarov

**Affiliations:** 1 Moscow Institute of Physics and Technology, Dolgoprudny, 141701 Russia; 2 Institute of Solid State Physics RAS, Chernogolovka, 142432 Russia; 3 Fundamental Physical and Chemical Engineering dep., MSU, Moscow, 119991 Russia; 4 Russian Quantum Center, Skolkovo, Moscow region 143025 Russia; 5 Department of Materials Science, MSU, Moscow, 119991 Russia; 6 Institute of Nanotechnology of Microelectronics RAS, Moscow, 119991 Russia; 7 National University of Science and Technology MISIS, Moscow, 119049 Russia; 8 Skobeltsyn Institute of Nuclear Physics, MSU, Moscow, 119991 Russia; 9 Solid State Physics Department, KFU, Kazan, 420008 Russia; 10 Faculty of Science and Technology and MESA+ Institute of Nanotechnology, 7500 AE Enschede, The Netherlands; 11 Department of Chemistry, MSU, Moscow, 119991 Russia; 12 Laboratoire de Physique et d’Etudes des Materiaux, LPEM, UMR-8213, ESPCI-Paris, PSL, CNRS, Sorbonne University, 75005 Paris, France; 13 Center for Fundamental and Applied Research, N. L. Dukhov All-Russia Research Institute of Automatics, 127055 Moscow, Russia

**Keywords:** Nanowires, Superconducting properties and materials

## Abstract

We examine the influence of superconductivity on the magneto-transport properties of a ferromagnetic Ni nanowire connected to Nb electrodes. We show experimentally and confirm theoretically that the Nb/Ni interface plays an essential role in the electron transport through the device. Just below the superconducting transition, a strong inverse proximity effect from the nanowire suppresses superconducting correlations at Nb/Ni interfaces, resulting in a conventional anisotropic magneto-resistive response. At lower temperatures however, the Nb electrodes operate as superconducting shunts. As the result, the magneto-resistance exhibits a strongly growing hysteretic behavior accompanied by a series of saw-like jumps. The latter are associated with the penetration/escape of individual Abrikosov vortices that influence non-equilibrium processes at the Nb/Ni interface. These effects should be taken into account when designing superconducting quantum nano-hybrids involving ferromagnetic nanowires.

## Introduction

Magneto-transport and magneto-dynamic properties of ferromagnetic (F) nanowires (NWs) currently attract a great interest due to their strong magnetic anisotropy and various size and spin effects^1,2^. These phenomena make F-NWs promising for applications in functional hybrid nano-devices, such as superconducting phase inverters, spin gates, magnetic memory elements^3–9^, etc. Usually, F-NWs are integrated into electronic circuits in which they are connected to normal-metal electrodes^10–18^. Magneto-resistive (MR) effects in such conventional circuits are dominated by the so-called anisotropic magneto-resistance (AMR), due to the anisotropy of the spin-orbit scattering in F-NWs^19^. As a result, the MR of the F-NW depends on the angle between the current and the magnetization, thus enabling one to correlate the magnetic state of the F-NW to its resistance^12–14,17,19^.

Recently a qualitatively new type of circuit has emerged, in which F-NWs are bonded to superconducting (S) electrodes^18,20^. In ref.^18^ an outstandingly long-range proximity phenomenon was reported: a Josephson effect between two W banks linked together by a 0.6 *μ*m long ferromagnetic Co-NW. In general, this domain of activity is motivated by a search for novel quantum properties of F-NW-based superconducting hybrids. Here we study the longitudinal MR of a S/F-NW/S hybrid device made of Ni-NW bonded to Nb superconducting electrodes. We demonstrate that below the superconducting transition temperature of Nb electrodes, the MR of the device is heavily dominated by the phenomena occurring at the Nb/Ni interface, where the superconducting Nb-electrodes operate as electric shunts of the Ni-NW. As a result, a hysteretic MR is observed on the top of a conventional AMR response. We also observed a series of quasi-periodic saw-like behavior in the MR signal, a direct consequence of the penetration of individual magnetic flux quanta - Abrikosov vortices from Nb/Ni interface into the superconducting electrodes. We suggest a model that explains the observed phenomena and the unusually high sensitivity of our S/F-NW/S device to the magnetic flux.

## Results and Discussion

### Resistivity near the superconducting transition

**Figure 1:**
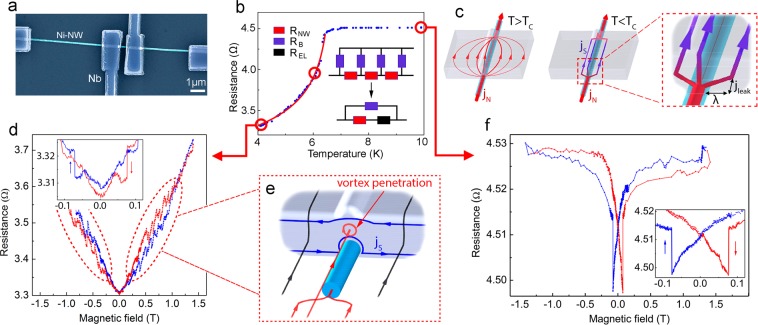
(**a**) Scanning Electron Microscopy image of the studied S/F-NW/S structure: Ni nanowire is coupled to four planar Nb electrodes. In the 4-probe measurements left and right electrodes serve for the current injection, and the two middle electrodes as voltage contacts. In the 2-probe experiments the middle electrodes are used as both voltage and current contacts. (**b**) Blue data points: *R*(*T*) dependence measured at *I*_*sam*_ = 30 *μ* A with the 4-probe scheme. Red dashed line: a fit using the effective resistance model. Sketch: the effective resistance circuit of the of the Nb/Ni-NW interface (see in the text). (**c**) Sketches of the current distribution in the overlapping region between Ni-NW and Nb voltage electrodes in the 4-probe experiment (see Supplementary Materials). Left - in the normal state of the Nb layer; center - in the superconducting state; right: a zoomed sketch of the current distribution at the edge of Nb/Ni interface. The superconducting current lines are depicted in blue, the normal current lines are in red. (**d**,**f**) Magneto-resistance *R*(*H*) measured at *T* = 10 K and 4.2 K, respectively. Insets: hysteresis loops due to the Ni-NW magnetization switching at ±0.076 T. Red dashed ellipses at (**d**) point to specific regions in *R*(*H*) where the Abrikosov vortex penetration causes a series of peaks in the magneto-resistance. (**e**) A sketch of the vortex penetration into Nb-electrodes at the Nb/Ni interface in the vicinity of the electrode’s edges (see in the text).

The resistance measured with 4-probes has a temperature dependence presented with blue dots in Fig. 1b. The resistance remains almost constant down to 6.7 K. Below this temperature it rapidly lowers but does not undergo a full transition to the superconducting state; the resistance remains relatively high even at 4 K. Additional 2-probe measurements confirmed (see Supplementary Materials) that the superconducting transition in Nb takes place at *T*_*C*_^*Nb*^ = 8.3 K, as expected. However, due to a strong inverse proximity effect from the F-NW the superconductivity of Nb electrodes near the Nb/Ni interface is suppressed; the resistance *R*_*El*_ of the Nb/Ni interface remains almost unchanged. Only at a significantly lower temperature *T*_*C*_ ≃ 6.7 K the interface resistance starts to decrease, witnessed by a major drop of the 2-probe resistance (see Supplementary Materials). The total resistance of the studied device comprises the resistance *R*_*Ni*_ of the Ni-NW itself, and the two Nb/Ni interface resistances *R*_*El*_. In the next subsection we evaluate the total resistance of the device and the temperature dependence of *R*_*El*_.

### Current flow at the Nb/Ni interface

The current flow in the overlapping Nb/Ni regions strongly depends on the state, normal or superconducting, of Nb-layer. In the normal state of Nb, the current distribution can be found from the solution of the Laplace equation for a scalar potential. This distribution is schematically shown on the left panel of Fig. 1c. Below the “effective” critical temperature *T*_*C*_ ≈ 6.7 K, at which the superconducting correlations in Nb start to affect the resistance near Nb/Ni interface, the current density is substantially redistributed (central panel in Fig. 1c). In the vicinity of the Nb/Ni interface the normal current is converted to supercurrent. This conversion takes place essentially in a narrow region at the inlet edge of the overlapping region (right panel in Fig. 1c). In the overlapping region, the current flowing through the NW splits, a part of which running into the superconducting Nb, which works as a superconducting shunt. There is no normal current flow in the central part of Ni-NW. Finally, an inverse conversion from supercurrent to the normal current takes place at the outlet edge of the overlapped region. As a result, the edge regions give an essential contribution to the interface resistance *R*_*EL*_, since both normal and supercurrent coexist.

In the 4-probe method the voltage difference is measured between two superconducting parts of Nb shunts. Considering two identical current conversions, one at the outlet edge of the first voltage lead and other at the inlet of the second one, the measured resistance *R* = *V*/*I* is:

1 $$R={R}_{Ni}+2{R}_{EL},$$

where the resistance of the Ni-NW is:

2 $${R}_{Ni}=\frac{4{l}_{NW}{\rho }_{Ni}}{\pi {d}^{2}}.$$

here, *l*_*NW*_ = 380 nm is the distance between middle Nb/Ni electrodes, *ρ*_*Ni*_ is Ni resistivity, *d* = 107 nm is the diameter of the nanowire, and *R*_*EL*_ is the effective resistance of one Nb/Ni electrode.

To find *R*_*EL*_ we subdivide the inlet and outlet areas of the Nb/Ni film in small blocks of a length Δ*l*. The Ni-part of this block has resistance

3 $${R}_{NW}=\frac{4{\rho }_{Ni}\Delta l}{\pi {d}^{2}}.$$

These Ni-parts connect to the superconducting Nb-shunt via an effective resistance of the current conversion area,

4 $${R}_{B}=\frac{{\rho }_{B}}{\Delta l\,\pi d},$$

where *ρ*_*B*_ is the specific Nb/Ni interface resistance.

The resistance of the inlet and outlet areas of the Nb/Ni electrodes *R*_*EL*_ can then be modeled as semi-infinite chain of resistances *R*_*B*_ and *R*_*NW*_, as represented in the inset of Fig. 1b. In this scheme the upper wire represents Nb-shunt. As a result, the total resistance *R*_*EL*_ is equal to the resistance of *R*_*B*_ and the sum *R*_*NW*_ + *R*_*EL*_ connected in parallel, as it is schematized in the inset of Fig. 1b:

5 $$\frac{1}{{R}_{EL}}=\frac{1}{{R}_{B}}+\frac{1}{{R}_{NW}+{R}_{EL}}.$$

From (5) we get

6 $${R}_{EL}={R}_{NW}(-\frac{1}{2}+\sqrt{\frac{1}{4}+\frac{{R}_{B}}{{R}_{NW}}}).$$

In the limit Δ*l* → 0 from expression (6) we finally have

7 $${R}_{EL}=\frac{2\sqrt{{\rho }_{Ni}{\rho }_{B}}}{\pi {d}^{3/2}}.$$

We now evaluate the temperature dependence of the interface resistivity *ρ*_*B*_. Due to the inverse proximity effect the superconducting gap of Nb is strongly suppressed at Nb/Ni interface; inside the Nb electrode it is restored to its bulk value only at a distance of the order of the temperature-dependent Ginsburg-Landau coherence length *ξ*_*GL*_(*T*) = (*π*/2)*ξ*_*Nb*_(1 − *T*/*T*_*C*_)^−1/2^, where *ξ*_*Nb*_ = (*ℏD*/2*πk*_*B*_*T*_*C*_)^1/2^, *D* is the diffusion coefficient. On a microscopic level, the conversion of the normal current into the superconducting current occurs due to Andreev reflection of quasiparticles from the wall of the potential barrier formed by the coordinate-dependent gap in the spectrum of the elementary excitations of Nb. As a result, the normal quasiparticles carrying the normal current are trapped in the region of the order of *ξ*_*GL*_(*T*) in the vicinity of Nb/Ni interface. This allows one to estimate the temperature dependence of the interface resistivity:

8 $${\rho }_{B} \sim \xi (T) \sim {\xi }_{Nb}{\mathrm{(1}-T/{T}_{C})}^{-\mathrm{1/2}}\mathrm{.}$$

In more advanced models^21–23^ the interface resistance has the temperature dependence ${\rho }_{B} \sim {(1-T/{T}_{C})}^{-\alpha }$, where *α* is indeed close to 0.5; its precise value is decided by characteristic quasiparticle energy relaxation processes in the superconductor. As a precise determination of the proportionality coefficient in the expression (8) is a difficult task, we combined (7) and (8) to *R*_*EL*_ = 0.5*A*(1 − *T*/*T*_*C*_)^−1/4^, and used the following expression to fit the observed temperature dependence of the total resistance:

9 $$R(T)={R}_{Ni}+A{(1-T/{T}_{C})}^{-1/4},$$

where *R*_*Ni*_ and *A* are the fitting parameters. This expression follows directly from (1) and explicitly takes into account the temperature dependence of the *R*_*EL*_ in (1). The best fit (red line in Fig. 1b) has been achieved with *R*_*Ni*_ ≈ 1.8 Ω and *A* ≈ 1.2 Ω. The first value fixes the resistivity of our Ni-NW to *ρ*_*Ni*_ ≈ 4.26 *μ*Ω cm, in agreement with the results of previous experiments on Ni-NW based structures measured at the liquid helium temperatures^16,24^.

In fact, the fitting parameters correspond to independent physical quantities. The first of these parameters, *R*_*Ni*_ is fully determined by the geometry of the nanowire and by its resistivity. The second fitting parameter *A*, according to Eqs (7 and 8) also depends on the boundary resistance of Nb-Ni interface. The magnitude of this parameter determines the length of conversion of a normal current into a supercurrent.

We can evaluate the characteristic scale *l*_*leak*_ of the current leakage into Ni wire from the Nb/Ni electrode. It can be done from (3) and (4) by equating resistances *R*_*NW*_ = *R*_*B*_, at which *l*_*leak*_ ≈ Δ*l*. We get:

10 $${l}_{leak}\approx {R}_{EL}\pi {d}^{2}/4{\rho }_{Ni}\approx 160\,nm,$$

at *T* = 4.2 K, that is an order of magnitude smaller the width of Nb electrodes visible in Fig. 1a.

The agreement of the fit with the experimental data confirms the assumption of the model that conversion of a normal current to supercurrent is mostly due to Andreev reflection processes at Nb-Ni interface, while the processes of high-energy quasiparticle relaxation are negligible.

### Magneto-transport: general behavior

Small length scale of conversion at the edges of the overlapping regions, of a normal current flowing in Ni-NW into a supercurrent in the Nb-shunt has important consequences for the magneto-transport properties of the device. The longitudinal magneto-resistance *R*(*H*) signals measured at 4.2 K (*T* < *T*_*C*_) and at 10 K (*T* > *T*_*C*_) are presented in Fig. 1d,f, respectively.

**Figure 2:**
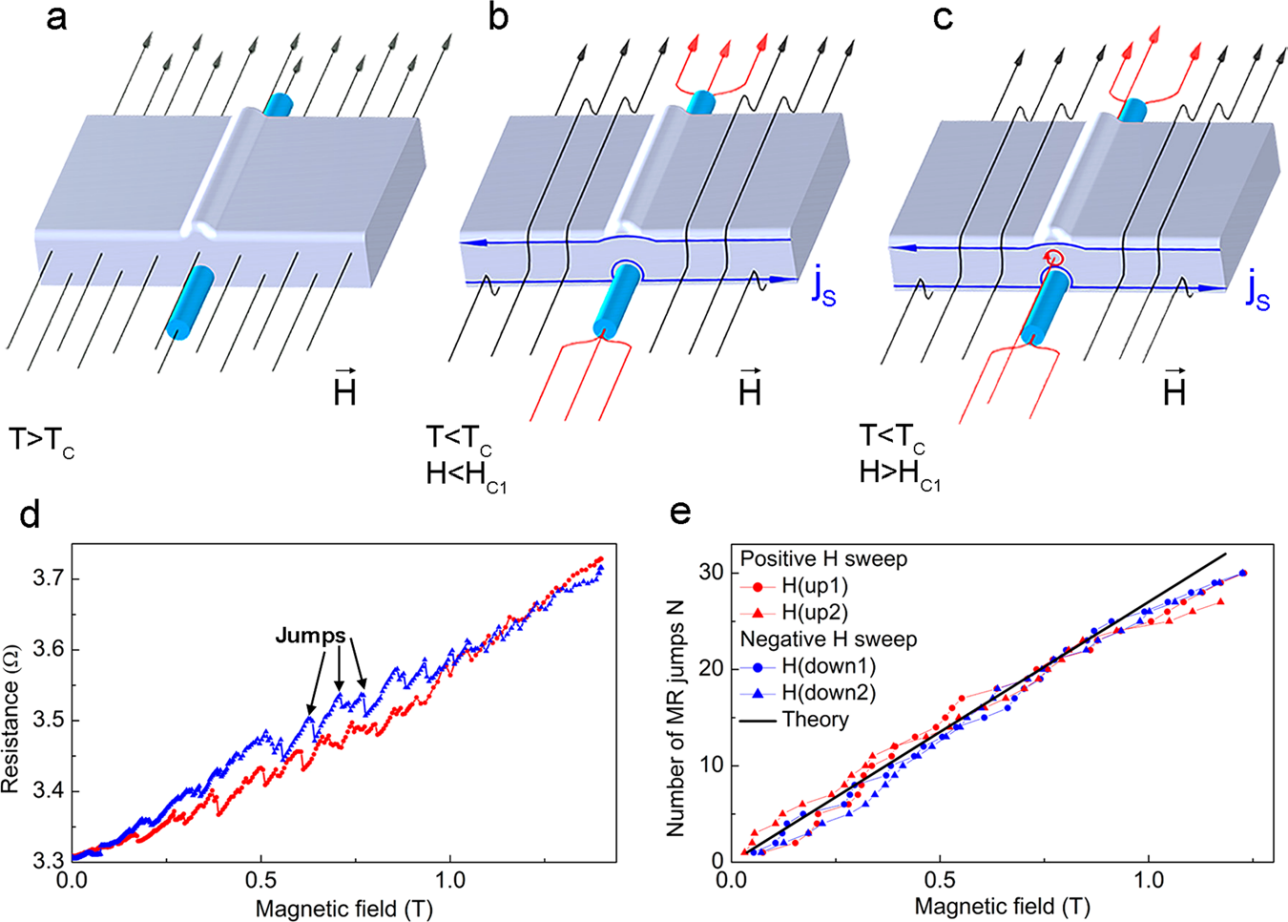
Device behavior at different external magnetic fields and temperatures. (**a**) *T* > *T*_*C*_, the magnetic field (presented by a series of black arrows) uniformly enters the sample. There is no screening current. (**b**) *T* < *T*_*C*_, at *H*_*c*_ < *H* < *H*_*c*1_ the magnetic field is expelled owing to screening Meissner currents (blue arrows) flowing along the edges of Nb-electrodes; it is focused in the nanowire. The screening current flow is strongly modified near Nb/Ni-interface. (**c**) An Abrikosov vortex penetrates inside Nb-electrode from Nb/Ni interface. (**d**) Enlarged picture of resistance jumps, marked by arrows, on *R*(*H*) dependence at *T* = 4.2 K, these jumps are almost equidistant. (**e**) Statistics of the magneto-resistance jumps visible in Figs 1d and 2d as a function of the applied field changing in different sweep directions.

When Nb-electrodes are in the normal state, *T* > *T*_*C*_, the magnetic field uniformly enters the sample, Fig. 2a. *R*(*H*) follows a conventional AMR^12,14,19,25^. Upon the magnetic field cycling *R*(*H*) shows a hysteresis due to the Ni-NW magnetization switches at a coercive field *H*_*c*_ = ±0.076T. The magneto-resistance does not exceed 0.7% in the entire field range 0–1.5T. At *H* = *H*_*c*_ *R*(*H*) jumps are Δ*R*_*M*_ ~ 0.015 Ω, that is about 3% of the average resistance (inset in Fig. 1f).

At *T* = 4.2 K, when the superconducting correlations from Nb reach Nb/Ni interface, *T* < *T*_*C*_ < *T*_*C*_^*Nb*^, the magneto-resistive behavior of the device changes drastically, Fig. 1d. *R*(*H*) grows rapidly and almost linearly from *R* = 3.33 Ω to *R* = 3.73 Ω, that is about 11% of variation in the entire field range 0–1.5 T. *R*(*H*) shows a hysteretic field response; the difference in the resistivity between *R*(*H*) branches measured in ascending and descending magnetic fields reaches ≃0.05 Ω at *H* = 0.7 T. Importantly, *R*(*H*) curves are saw-shaped (in the regions marked by dashed ellipses in Fig. 1d) with rather regular resistivity jumps by up resistivity drops of up to Δ*R*_*V*_ ≃ 0.05 Ω. The amplitude of these drops is larger than the whole AMR observed at 10 K. In addition, AMR jumps of Δ*R*_*M*_ ≃ 0.01 Ω associated with magnetization of the F-NW and AMR phenomenon remain (see inset in Fig. 1d). Notice however, that the relative AMR *R*(*H*) variation at *H*_*c*_ is smaller as compared to the high temperature behavior, Fig. 1f.

Since the magnetic switching of the Ni-NW leads to only tiny variations of *R*(*H*) (see inset in Fig. 1d), the main MR features in Fig. 1d should be intimately related to phenomena occurring near Nb/Ni interface. The resistance of these regions is mostly determined by non-equilibrium processes of the conversion of the normal current to supercurrent and back^21–23^. In our estimates, at *T* = 4.2 K and *H* = 0 the conversion takes place on the length ≈160 nm (10), resulting in the interface resistance of 1–3 Ω. The latter depends significantly on the suppression of the superconducting correlations in the vicinity of the NW.

When an external magnetic field is applied along the NW, the Meissner screening currents *j*_*S*_ start to circulate (blue lines in Fig. 2b). In an increasing field they progressively suppress the superconducting correlations in the vicinity of the Nb/Ni interface and provoke a growth of the conversion length. As a result, the interface resistance increases. This explains the general increase in *R*(*H*) behavior seen in Fig. 1d.

### Magneto-transport: signatures of Abrikosov vortices

We now discuss the puzzling features - sudden resistance jumps observed in the *R*(*H*) curves in the regions marked by ellipses in Fig. 1d. The zoom of this curve is demonstrated on Fig. 2d. These jumps are related to the Abrikosov vortex penetration into the device, as we discuss below. When the applied field exceeds the first critical field *H*_*c*1_ of Nb, a mixed superconducting state with Abrikosov vortices becomes energetically favorable. As Meissner screening currents *j*_*S*_ also increase with the magnetic field, they weaken the superconducting order at the edges of Nb and facilitate the vortex penetration. The weakest edge region from which the vortices can penetrate is located close to Nb/Ni interface where the superconducting order is further suppressed due to the proximity with Ni-NW (Fig. 2b).

The penetration of a single vortex into Nb partially relaxes both the magnetic energy and the screening Meissner currents^26^. The latter effect is especially important near the Nb/Ni interface, due to a peculiar geometry of the device (see Fig. 2c). A sudden reduction of the screening currents partially restores the superconducting correlations near Nb/Ni interface and reduces the conversion length. A drop Δ*R*_*V*_ in the interface resistance occurs. Upon further increase of the field, the screening currents start to grow again until another vortex enters. The process continues and results in a series of sudden resistance jumps observed in *R*(*H*), Figs 1d and 2e, each jump witnessing for the penetration of one single quantum vortex into Nb-electrode.

As seen in Figs 1d and 2e the jumps Δ*R*_*V*_(*H*) appear nearly periodic; they indeed can be associated to individual vortex penetration. It is not clear however, if the device detects all vortices one by one. A simple estimate of the total number of vortices penetrated into the sample at a field *H* can be done and compared to the statistics of the observed events, presented in Fig. 2e. Due to the device geometry, the area *S* into which Abrikosov vortices penetrate from a given injection point is of the order of $S \sim {h}^{\ast }\times {\lambda }_{Nb}$ = 1.4 × 10^4^ nm^2^, where ${h}^{\ast }\simeq h-2{\xi }_{GL}\simeq 180\,{\rm{nm}}$ is the effective thickness, and ${\lambda }_{Nb}\simeq 80\,{\rm{nm}}$ is the London penetration depth in our granular Nb-film at *T* = 4.2 K. At a field *H* there should be around $N=(S/{\Phi }_{0})\times H \sim$10–20 × *H*[*T*] Abrikosov vortices (in this estimation Φ_0_ is the magnetic flux quantum, the diamagnetic screening is neglected). Therefore, if the two electrodes are perfectly identical, the vortices penetrate into them simultaneously, and one would expect to count 10–20 jumps per Tesla. If the contacts are slightly different, then one Δ*R*_*V*_ jump corresponds to the penetration of either one or two vortices, and the expected number of events per Tesla could be 10–40. The black line in Fig. 2e which matches the event statistics has the slope 28 × *H*[*T*], within the estimated numbers. This witnesses for the capacity of the device to detect individual quantum vortices.

Despite the quantum origin of the vortices and the resulting flux quantization, the resistance jumps Δ*R*_*V*_ are not regular: they have different amplitudes and a slightly aperiodic occurrence in the rising/lowering field. There are several reasons for such “irregular” behavior. Indeed, once entered into Nb-electrode, the vortices leave the interface area. Their equilibrium location in Nb depends on the presence of other vortices in the neighborhood, surface and bulk defects etc. The resistance jumps are stronger for vortices propagated far away from the interface, and smaller for vortices remained in the vicinity. The same explanation applies to the hysteresis in *R*(*H*), and can be satisfactorily interpreted with the Bean critical state model^27,28^. Within this scenario, the end of hysteresis on *R*(*H*) curve in Fig. 1d at $H\simeq 1{\rm{T}}$ corresponds to the so-called superconducting irreversibility field^29–31^. Other than the amplitude Δ*R*_*V*_ of the jumps, their periodicity in the field is also affected by the presence of other vortices inside the device, defects etc. Finally, we should bear in mind that the 4-probe resistance comprises the sum of the inlet and outlet Nb/Ni interface resistances that were considered equal in (1). In real devices, the contacts have slightly different characteristics, resulting in vortices penetrating at different values of the external field. The drops in the total *R*(*H*) signal represent a sum of the two series of events.

### Magneto-transport: peak effect near the normal state of the interface

**Figure 3:**
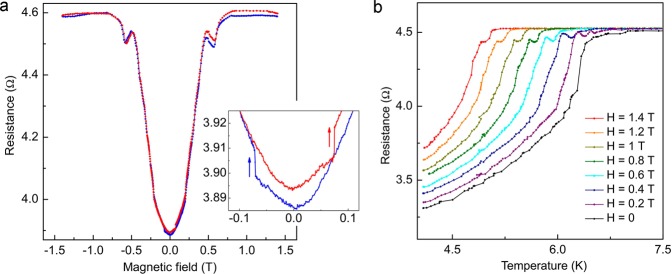
(**a**) Magnetoresistance *R*(*H*) measured at *T* = 6 K. The inset shows magnified *R*(*H*) curves in the vicinity of *H*_*c*_. Red and blue colors correspond to positive and negative sweep of the magnetic field. Arrows indicate direction of nanowire magnetization reversal. (**b**) Evolution of the temperature dependencies of resistance of the Ni nanowire for different applied magnetic fields. The peak effect close to *T*_*C*_ could be related to the spin accumulation at the Nb/Ni interface (see explanation in the text).

Figure 3a shows *R*(*H*) curves measured at *T* = 6 K, that is in the middle of the resistive transition (marked by a red circle in Fig. 1b). In the increasing magnetic field branch, the resistance grows rapidly and saturates at the normal state resistance value at $H\simeq \pm \,0.8\,{\rm{T}}$. This indicates that the field efficiently weakens the superconducting correlations at Nb/Ni interface and renders neighboring parts of the Nb-electrodes non-superconducting. The AMR jumps at *H*_*c*_ (inset in Fig. 3a) still exist but the hysteresis in *R*(*H*) curves vanishes. Also, Δ*R*_*V*_ jumps associated with the vortex penetration/escape are not detected anymore. The main reason is that, unlike at *T* = 4.2 K, the effective S/N boundary at which the Abrikosov vortices are formed and penetrate into superconductor is pushed far away from the actual Nb/Ni interface region deeper into Nb.

A puzzling peak is observed in the field interval $H\simeq \pm$(0.5–0.6) T at which *R*(*H*) drops by ~0.05 Ω. A similar drop in the vicinity of the transition to the normal state is observed on *R*(*T*) curves in the interval of several tenths of Kelvin, as shown in Fig. 3b for *R*(*T*) measured at various fields. This phenomenon is washed out at zero field *H* = 0 but is present at other fields.

The origin of this effect is the spin-polarization process in the nanowire which leads to spin accumulation at the boundary. While the current inside Ni-NW is spin-polarized, the supercurrent in superconducting Nb-electrode is spinless. That means that only spinless part of incoming current can be converted to supercurrent, leading to spin accumulation at the boundary^32,33^. As a result, part of current turns out to be redistributed from Nb into the Ni-nanowire, leading to increase of the total resistance. The amplitude of resistance peak depends on the rate of spin-flip processes inside NW. At *H* = 0 the presence of domain walls leads to increase in spin-less component which able to leakage into superconducting electrodes. At *H *> 0 the number of domain walls decreases and as the result the rate of spin-flip intensity rises, thus, the resistance drops appear.

## Conclusion

To conclude, we investigated experimentally and theoretically the electron transport properties of Ni-nanowires coupled to superconducting Nb-electrodes. We showed that the Superconductor-Ferromagnet interface plays an essential role in the magneto-resistive behavior of the device. Several effects related to the nano-scale phenomena at this interface were observed and explained. At temperatures just below the superconducting transition of Nb a strong inverse proximity effect from Ni-nanowire dominates the magnetic response, preserving a conventional anisotropic magneto-resistance measured at higher temperatures. At temperatures well below 6 K however, the superconducting order strengthens, and the Nb-electrodes play the role of superconducting shunts in the overlapped Nb/Ni areas of the device. This leads to a strong magneto-resistance that shows a well-pronounced hysteresis. The most intriguing feature is a series of jumps in the magneto-resistance related to the penetration of individual Abrikosov vortices through the Nb/Ni interface. At intermediary temperatures ~6 K the hysteresis and vortex-triggered magneto-resistivity jumps are washed out. Additional peaks appear in the vicinity of the critical transition. They are associated with non-equilibrium processes at the Nb/Ni interface. Similar peak features are observed on *R*(*T*) curves in the vicinity of critical temperature at non-zero fields. Importantly, the anisotropic magneto-resistance of the nanowire exists at all temperatures. This provides an opportunity to develop novel cryogenic hybrid nano-devices which take advantage of both anisotropic magneto-resistance and complex phenomena revealed at the Superconductor/Ferromagnet interface.

## Methods

Nickel nanowires of 100–150 nm in diameter were obtained by templated electro-deposition into a porous anodic alumina^34,35^, extracted, washed, and dispersed in heptane (see Supplementary Materials). Individual NWs were bonded to Nb-electrodes for 4-probe transport measurements, following the steps comprising a seeding onto a marked Si/SiO_2_ substrate, electron lithography, magnetron sputtering, and lift-off processes (see Supplementary Materials and^36^). A set of samples was fabricated with different distances between inner (voltage) electrodes and with NWs of different diameters. They showed a qualitatively similar behavior. The device we discuss in this work was made using a 107 nm thick Ni-NW. The SEM image of the sample is shown in Fig. 1a. The distance between the two voltage electrodes is 380 nm. Both 4-probe and 2-probe transport measurements were provided. In all measurements the applied current was *I*_*sam*_ = 30 *μ*A. A magnetic field up to 1.5T was applied in the direction parallel to the Ni-NW main axis.

## Supplementary information

Supplementary information
