# Supplementary material for: Anomalous magneto-resistance of Ni-nanowire/Nb hybrid system
Source: Sci Rep. 2019 Oct 9;9:14470. doi: 10.1038/s41598-019-50966-8 (PMC6785530; doi:10.1038/s41598-019-50966-8)
Supplement: Supplementary file 1 — Supplementary information [file 41598_2019_50966_MOESM1_ESM.pdf]

Supplementary information. Anomalous magneto-resistance of Ni-nanowire / Nb hybrid system

O.V. Skryabina, S.N. Kozlov, S.V. Egorov, A.A. Klimenko, V.V. Ryazanov, S.V. Bakurskiy, M.Yu. Kupriyanov, N.V. Klenov, I.I. Soloviev, A.A. Golubov, K.S. Napolskii, I.A. Golovchanskiy, D. Roditchev, V.S. Stolyarov

Electro-chemical preparation and characterization of Ni-nanowires

Nickel nanowires were obtained by templated electrodeposition. Porous anodic aluminum oxide (AAO) prepared by anodization of high purity Al (99.999%) at 120 V in 0.3 M $H_2C_2O_4$ was used as a template [1, 2]. The aluminum remained after the anodization was selectively dissolved in 4 M solution of Br_2 in methanol. In order to obtain oxide films with through hole channels the barrier oxide layer was removed by chemical etching in 3 M H_3PO_4 solution at room temperature with the electrochemical detection of pore opening moment [3]. Then a 300 nm thick gold layer serving as a current collector was deposited to the bottom of AAO film by magnetron sputtering. Nickel electrodeposition was carried out in three-electrode teflon electrochemical cell using electrolyte containing 0.6 M $NiSO_4$, 0.1 M $NiCl_2$, and 0.3 M H_3BO_3 at room temperature and constant deposition potential of -0.9 V versus saturated (KCl) Ag/AgCl reference electrode connected to the cell via Luggin capillary. A platinum wire ring was used as a counter electrode and was set in parallel to the surface of the working electrode. The electrodeposition was stopped when the metal reached the external surface of the AAO template. In order to extract Ni nanowires the oxide matrix was selectively dissolved. For this purpose Ni/AAO nanocomposite was placed for 12 hours into the oxidant-free alkaline solution containing 3 M NaOH, 0.4 M glucose, and a few drops of methylene blue solution. Glucose is easily oxidized in alkaline solution by dissolved oxygen, which leads to a decrease in O_2 concentration. Methylene blue acts as a catalyst of this process. Thus, the addition of the above-mentioned compounds prevents the oxidation of Ni nanowire surface by oxygen. It is worth noting that in the absence of oxygen the methylene blue transforms into transparent form under the influence of glucose. This color change can be used as an indicator of oxygen concentration in the solution. After complete dissolution of the oxide matrix the samples were washed by decantation with fresh oxidant-free alkaline solution, five times with deionized water, and then five times with isopropanol. Finally nanowires were dried in vacuum and suspended in heptane by ultrasonication.

According to statistical analysis of scanning electron microscopy (SEM) images, the diameter of Ni nanowires in the suspension varies from 100 to 150 nm due to the dispersion of pore diameter in AAO template. At that, the variation of diameter along the length of a single nanowire does not exceed 10 nm. Transmission electron microscopy (TEM) and selected area electron diffraction (SAED) revealed polycrystalline structure of the nanowires (see Figs. 1a,b). The average size of individual single crystal grains is close to the diameter of

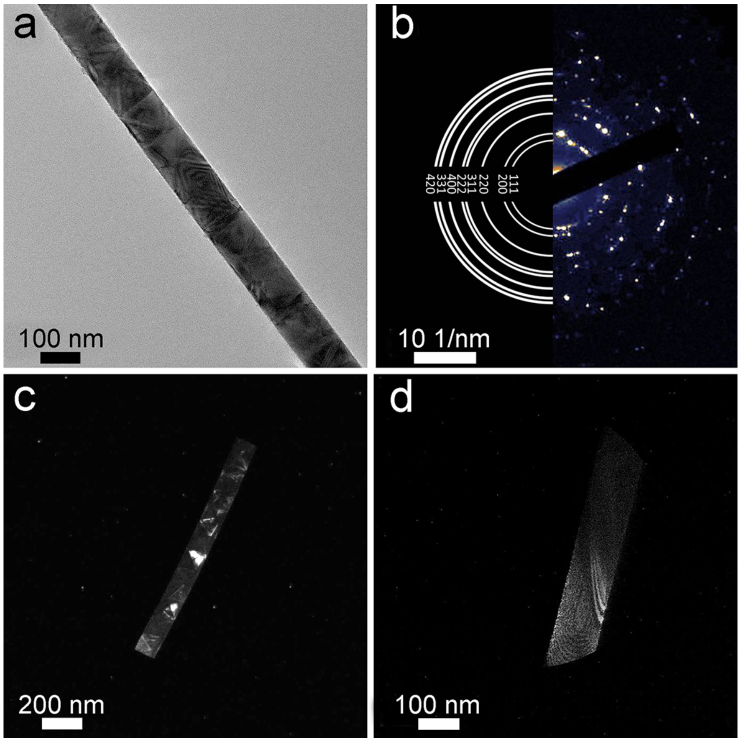

Figure 1: TEM (a) and SAED (b) data for single Ni nanowire. Dark-field TEM images of the single Ni nanowire are shown in panels (c) and (d).

NWs (Fig. 1c), but in some cases it can exceed 500 nm (Fig. 1d). Native oxide with the thickness of 4 nm covers the surface of Ni nanowires and protects the metal from further oxidation. It is worth noting that the composition and structure of the nanowires remains constant during their storage in suspension during several months.

Hybrid nanostructure preparation

Individual NWs were 4-probe bonded for transport measurements using successive seeding of Ni NWs onto marked Si/SiO_2 substrate, electron lithography, magnetron sputtering of Nb film, and lift-off processes. Prior to the deposition of 250 nm thick Nb electrodes, the samples were etched in the argon plasma in order to achieve a clean electron-transparent Nb/Ni interface. Note, that the thickness of Nb is substantially larger than the London penetration depth λ_L.

A set of samples was fabricated with different distance between voltage electrodes and different diameters of NWs. All samples showed a qualitatively

similar properties.

Electron transport measurements

The electronic and MR transport measurements were performed in He-4 cryostat equipped with 1.4 T superconducting solenoid, at temperatures down to 4.2 K in 2-probe and 4-probe geometries. The magnetic field was applied parallel to the main axis of the NW. All measurement lines were equipped with low temperature RC filters.

The results of the 2-probe measurements are presented in Fig. 2a. In this figure a strong inverse proximity coupling at the Ni-Nb interface is evidenced by a heavily suppressed superconductivity of Nb electrodes near the Nb/Ni interface. The superconducting transition temperature of the deposited Nb is $T_C^{Nb} = 8.3$ K: A small drop in the 2-probe resistance at 8.3 K is indeed observed (indicated by an arrow). However, in the vicinity of Nb/Ni-NW interface the superconducting transition in Nb is shifted down to $T_C \simeq 6.7$ K, as follows from the major drop of the 2-probe resistance. At that, the change in 4-probe resistance at the transition is observed but is marginal, invisible in Fig. 2b.

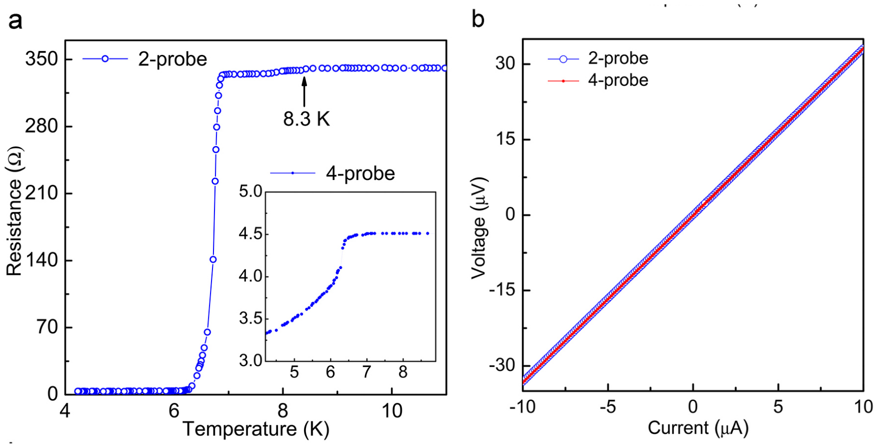

Figure 2: (a) $R(T)$ dependence measured at $I_{sam} = 30$ μA in 2-probe scheme. Transition temperature of superconducting Nb at $T = 8.3$ K is indicated with arrow. The inset shows the $R(T)$ dependence measured in 4-probe geometries. (b) $I(V)$ characteristics measured at $T = 4.2$ K in both 2-probe and 4-probe schemes.

A high electron-transparency of the Nb/Ni interface is evidenced by a perfect overlap of current-voltage characteristics acquired in 2- and 4-probe measurements at $T = 4.2$ K (Fig. 2b). These $I(V)$-characteristics imply that at this temperature the net resistance of the whole structure measured between the voltage leads is $R = 3.3$ Ω. This comprises the resistance R_{Ni} of the Ni-NW itself, and the two Nb/Ni interface resistances R_{El} (see subsection "Current flow

at the Nb/Ni interface" in the main text).

Derivative data at 6 K (Fig. 3(a) in the main text) are demonstrated at Fig. 3. One can observe periodic events on the derivative that should be related to flux quantization. The first event occurs at 0.2 T and the Δ_H between events increases gradually with magnetic field (0.04 T between 1 and 2, and 0.9 between 3 and 4). Gradual increase of Δ_H with H may be a result of geometrical/Bean-Livingston barriers acting against vortex penetration together with the Meissner screening.

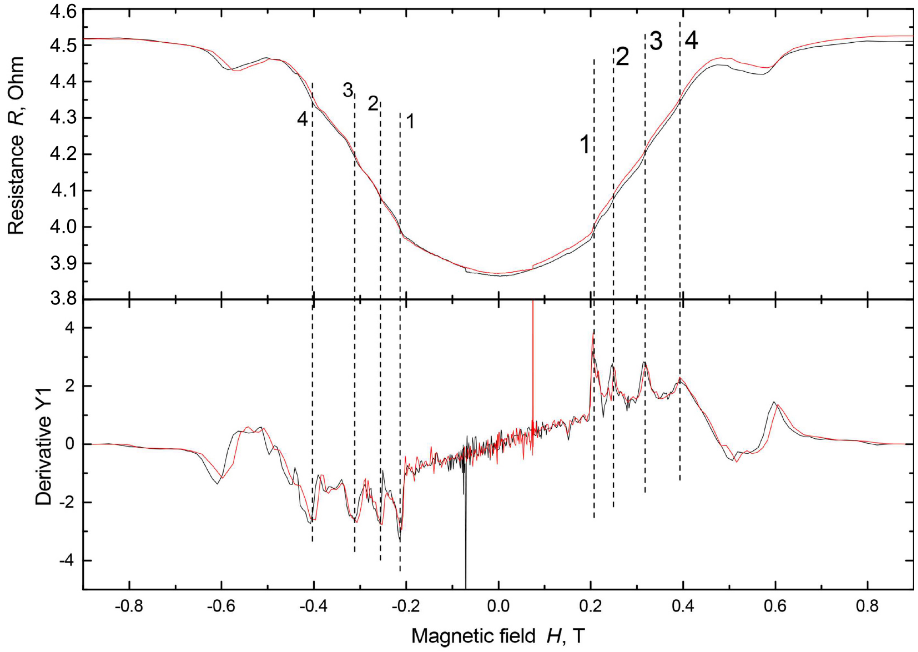

Figure 3: Derivative data R(H) at 6 K.

References

[1] Lee, W., Ji, R., Gosele, U., Nielsch, K. Fast fabrication of long-range ordered porous alumina membranes by hard anodization. *Nature Materials* **5**, 741–747 (2006).

[2] Napolskii, K. S. et al. Tuning the microstructure and functional properties of metal nanowire arrays via deposition potential. *Electrochim. Acta* **56**, 2378 (2011).

[3] Lillo, M., Losic, D. Pore opening detection for controlled dissolution of barrier oxide layer and fabrication of nanoporous alumina with through-hole morphology. *J. Membrane Sci.* **327**, 11 (2009).
